# Implantable antennas for biomedical applications: a systematic review

**DOI:** 10.1186/s12938-024-01277-1

**Published:** 2024-08-29

**Authors:** Archana Mohan, Niraj Kumar

**Affiliations:** School of Electronics Engineering, Vellore Institute of Technology, Chennai, Tamilnadu India

**Keywords:** Implantable medical device (IMD), Implantable antennas, Biocompatibility, Miniaturization, Pacemaker, Intracranial pressure monitoring, Capsule endoscopy, Retinal prostheses

## Abstract

This review presents an in-depth examination of implantable antennas for various biomedical purposes. The development of implantable antennas, including their designs, materials, and operating principles, are introduced at the beginning of the discussion. An overview of the many kinds of implantable antennas utilized in implantable medical devices (IMDs) are presented in this study. The article then discusses the important factors to consider when developing implantable antennas for biomedical purposes, including implant placement, frequency range, and power needs. This investigation additionally examines the challenges and limitations encountered with implantable antennas, including the limited space available within the human body, the requirement for biocompatible materials, the impact of surrounding tissue on antenna performance, tissue attenuation, and signal interference. This review also emphasizes the most recent advances in implanted antenna technology, such as wireless power transmission, multiband operation, and miniaturization. Furthermore, it offers illustrations of several biomedical uses for implantable antennas, including pacemaker, capsule endoscopy, intracranial pressure monitoring, retinal prostheses, and bone implants. This paper concludes with a discussion of the future of implantable antennas and their possible use in bioelectronic medicine and novel medical implants. Overall, this survey offers a thorough analysis of implantable antennas in biomedical applications, emphasizing their importance in the development of implantable medical technology.

## Introduction

Implantable antenna technology is a contemporary movement in biomedical applications. The implantation of antennas is used in biomedical diagnosis, therapy, and biotelemetry. Antennas are inserted into the human body for use in electromagnetic wave-based biomedical applications. Gathering patient data and wirelessly communicating it to the base station is the goal of the implanted device within the body area. The trend of implantation started in the 1960s and the first biomedical implantable device was a cardiac pacemaker, to control irregular heart rhythms [1]. The use of an implantable antenna (radiators) for cancer detection, to generate confined bottomless heat for cancer treatment without overheating the surface is important in situations where the implantation of the antennas is applied [2]. Since the population has progressed and public awareness about health has increased, IMDs have become a rising technology. Currently, several drug diffusion systems, implantable defibrillators, and implantable pacemakers are widely used in the biomedical field [3]. Implantable antennas are used to calculate various important parameters of the human body such as continuous glucose monitoring [4], temperature measurements [5], and heart function detection [6].

Implantable antennas are obligatory for wirelessly transmitting data from infix devices to exterior devices. Medical implants are used to identify, diagnose and monitor the medical circumstances of patients. Another major application of the implantable antenna is biotelemetry, and much research progress is working toward medical implants that are accomplished in appropriate frequency bands with minimum interference [7–16]. The transmission of the data is carried out in a wireless medium, so security is an important concern when transferring the IMD data [17].

The radio frequency allocation for medical implants varies from region to region according to the regulatory authority. In the United States, frequency band allocation is divided into short-range and long-range bands on the basis of the Federal Communication Commission (FCC) [18–20]. In Europe, spectrum allocation is split into active medical implants and associated peripherals and the medical data acquisition range is based on the European Communication Committee (ECC) [20, 21]. Compared with constructing an antenna operating in free space, constructing an antenna in body application is a difficult task [22]. The subsequent frequency bands are used in biotelemetry applications:

- Medical implant communication services (MICSs): 402–405 MHz [23, 85].
- Medical device radio communications service (Med Radio): 401–406, 413–419, 426–432, 438–444, and 451–457 MHz [24, 62].
- Industrial scientific and medical (ISM) band: 433.05–434.79, 902–928, 2400–2483.5, 5725–5850 MHz
- WMTS bands (608–614 MHz, 1395–1400 MHz, 1427–1432 MHz) [25].
- Ultra-wide band (UWB): 3100–10600 MHz [26].
- Medical body area network (MBAN): 2360–2400 MHz [27, 28].

The higher operating frequencies retain narrow wavelengths that lead to a reduction in the dimensions of the antennas. For better data communication, antennas operating at higher frequency ranges with large bandwidths are needed, but they are affected by a greater decrease in the amount of tissue compared with the small frequency ranges [29, 42].

The frequency range from 3 to 5 GHz had 20 to 30 dB attenuation for every 2 cm of physiological material [30]. The communication speed is limited by the lower operating frequencies and the use of large dimensional antennas and bulky circuit elements, which increase the size of the implantable medical device [33].

**Fig. 1:**
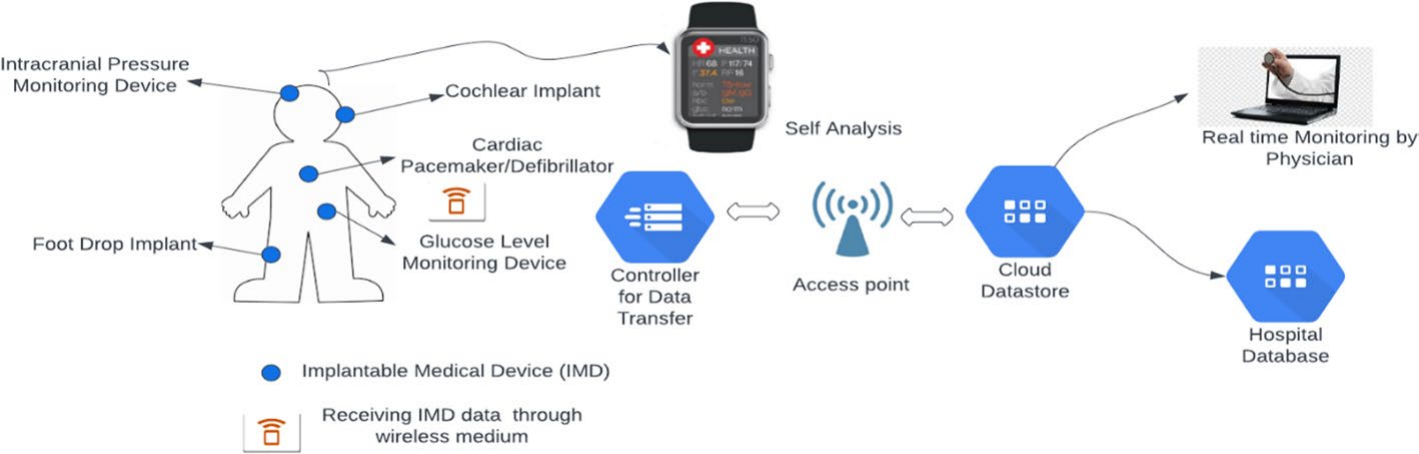
Model of an implantable medical device with biotelemetry [17]

Figure 1 shows a model of implantable medical devices with biotelemetry. Here some implantable medical devices are appropriately placed on different body parts. From the IMD, the data are collected, accessed via the cloud database, and sent to the concerned persons via a wireless medium.

### Significance of implantable antennas

*Real-time monitoring.* Implantable antennas make it possible to continuously monitor vital signs and physiological parameters, which can lead to early detection of medical conditions and prompt intervention.

*Enhanced mobility.* With wireless communication enabled by implantable antennas, patients can move around freely without being tethered to monitoring equipment, thereby improving their quality of life.

*Minimally invasive care.* These antennas reduce the need for frequent doctor visits and invasive tests, making procedures less invasive and more comfortable for patients. Additionally, they minimize the risk of infection.

*Remote healthcare and telemedicine.* Implantable antennas facilitate telemedicine and remote patient monitoring, making healthcare available to those in remote or underserved areas.

*Smart implants.* Implantable antennas are essential for smart implants, such as pacemakers, insulin pumps, and neural stimulators, allowing for precise control and fine-tuning of the devices.

## Strategies for designing implantable antenna

In contrast to outdated antennas, which operate in free space, implantable antennas, which are anchored in different body parts, must be esthetically acceptable in a variety of situations. The implantable antenna should satisfy the requirements of biocompatibility, miniaturization, patient safety, far-field gain, and low power consumption.

### Biocompatibility

The main design consideration of the implantable antenna is biocompatibility to prevent patient safety and avoid radiation to surrounding tissues. Furthermore, because human tissues naturally conduct electricity, human tissue antennas have the ability to make direct contact with the antenna metallization layer, which can cause a short circuit. The anticipation of undesirable short circuits and biocompatibility are more crucial for long-term implantation of the implantable antenna.

**Fig. 2:**
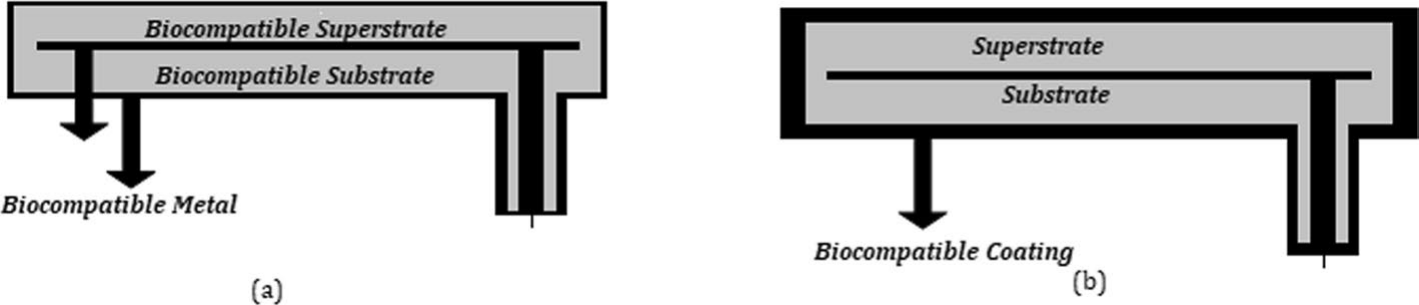
**a** Biocompatible superstrate. **b** Biocompatible coating

Biocompatibility is addressed via two methods: the use of a dielectric superstrate layer and the use of a biocompatible coating. The most commonly used approach is the use of a dielectric superstrate layer to separate the antenna metal radiator from biological tissue and conserve the biocompatibility of the implantable antenna [31–37]. Commonly used superstrate materials include Teflon (${\varepsilon }_{r}=2.1,\text{tan}\delta =0.001)$, MACOR (${\varepsilon }_{r}=6.1,\text{tan}\delta =0.005)$ and ceramic alumina (${\varepsilon }_{r}=9.4,\text{tan}\delta =0.006)$ [38–41]. Another approach is to shield the implantable material with a thin layer of biocompatible materials such as zirconia (${\varepsilon }_{r}=29,\text{tan}\delta \approx 0)$, PEEK (${\varepsilon }_{r}=3.2,\text{tan}\delta =0.01),$ and silastic MDX 4210 (${\varepsilon }_{r}=3.3,\text{tan}\delta \approx 0)$ [37, 42, 43]. Figure 2 shows the two methods of biocompatibility: Fig. 2a shows a biocompatible superstrate and 2b shows a biocompatible coating.

### Miniaturization

Miniaturization is one of the most important design considerations for implantable antennas. Recent advanced technologies in antenna fabrication have led to ultra-small implantable antennas. For example, cochlear implants and retinal prostheses are very small and are inserted inside the eyeball and auditory nerves. The geometries of conventional half-wavelength or quarter-wavelength antennas at the wave bands are assigned to the implanted devices, typically the MICS or Med radio bands. In this state, miniaturization is challenging. Some of the miniaturization techniques are listed below.

#### High-permittivity dielectric substrate/superstrate materials

Utilizing a substrate/superstrate made of a high-permittivity dielectric material is one of the coolest ways to achieve miniaturization because it condenses the effective wavelength and high resonant frequencies are shifted to lower resonant frequencies. A thicker superstrate increases the operating frequency of the implant and larger physical dimensions are required to enhance the resonance [44]. Some highly dielectric materials are listed in Table 1, as mentioned in the literature [45–49].

**Table 1:** Few of the materials used as substrates and superstrates

Material	Relative dielectric constant (${{\varvec{\varepsilon}}}_{{\varvec{r}}}$)
Rogers TMM10	9.2
Ceramic alumina	9.4
Rogers 3210/Rogers 6002/Rogers 3010/ARLON 1000	10.2
${MgTa}_{1.5}{Nb}_{0.5}{O}_{6}$	28

#### Extending the radiator’s current flow path

This method is used to calculate the overall size of the antenna; the longer the radiator's effective current path is, the larger the resonant frequency that could be shifted to a lower resonating frequency. Here, waffle-style, spiral and hook-slotted patches are utilized [48, 49]. In particular, meandering line radiators consist of zigzag or serpentine patterns to elongates the physical path of the radiator within a small footprint [50].

#### Employing shorting pins

By adding the shorting pin between the patch and ground planes, the necessary physical dimensions are reduced at the appropriate operating frequency. Shorting pins effectively enhance the bandwidth in both regular patches and quarter wave patches [38, 51–55].

#### Patch staking

By extending the radiator's current flow path, the antenna dimensions decrease when two radiating patches are mounted vertically [54, 55]. A metamaterial-based superstrate is added with antenna structure to enhance the axial ratio, bandwidth and gain [56].

#### Impedance matching

Impedance matching is achieved by loading methods, with the appropriate capacitance or inductance used to offset the frequency band and direction. Both capacitive and inductive loadings are employed to minimize the antenna size. Furthermore, the split-ring resonator (SRR) can be merged to achieve size reduction and impedance matching [35, 55].

### Patient safety

#### Specific absorption rate (SAR)

Patient safety is measured by the specific absorption rate (SAR—the rate of energy deposited per unit mass of tissue) parameter on the basis of IEEE regulations and guidelines used to prevent patient health [57]. Patient safety is related to the maximum permissible power applied to the implantable antenna. For example, the IEEE c95.1_1999 standard confines the SAR averaged over 1 g of tissue in the shape of a cube to less than 1.6 W/kg (${SAR}_{1g, max} \le 1.6 W/kg$) [56]. The ICNIRP elementary limitations are that the SAR averaged over 10 g of connecting tissue is less than 2 W/kg [59]. To correspond with the ICNIRP guidelines, the IEEE C95.1–2005 standard confines the SAR averaged over any 10 g of tissue in the shape of a cube to less than 2 W/kg (${SAR}_{10g, max} \le 2 W/kg$) [59, 60].

#### Temperature limit

Due to an electromagnetic field, the temperature of the body tissue is increased. The temperature of the surrounding tissue of the implantable antenna is not $1-2\,^\circ{\rm C}$ [35]. In some rectenna and pacemaker type applications, prolonged battery charging and rectifier (wireless power transfer) circuits increased the temperature [61].

### Gain in the far-field

Typically, medical implant communication systems (MICSs) consist of an external monitoring device positioned approximately two meters away from the implantable medical device (IMD) and an implantable medical device [62]. Both the recorded vital parameters and the real time monitoring of the body’s vital parameters are transmitted via medical telemetry lines. As a consequence, the external device has to improve the signal strength of the implantable antenna to a satisfactory level. In addition to patient safety, intrusion also restricts the permissible power required for implantable antennas. For example, in the MICS band, the effective radiated power (ERP) of the implantable antenna is restricted to – 16 dBm (25 μW), to avoid nearby service band interference. In addition to effective radiated power, SAR restricts the ability of the gain of the far-field gain to be sensed by the receiver antenna to achieve a consistent biotelemetry link. However, to increase the biotelemetry communication link distance, an implantable antenna with an enhanced gain is needed [63–74].

### Radiation efficiency

Radiation efficiency is a crucial factor for implantable antennas as it measures the antenna's effectiveness in converting input power into radiated electromagnetic energy. However, obtaining high radiation efficiency for implantable antennas is difficult because the surrounding biological tissues tend to absorb and weaken electromagnetic waves. The efficiency of EM energy transfer from dispersive tissues to free space is influenced by several frequency-dependent mechanisms. These include attenuation resulting from dielectric and conductive losses, reflection (mismatch) losses caused by impedance contrast, and physical restrictions on the radiation efficiency of electrically small sources within lossy media. The following methods are used to increase the radiation efficiency while maintaining the compact size of electrically small antennas: bandwidth reduction, and dielectric loading to the antenna structure by using a high-permittivity substrate and a superstrate [75–79].

### Low power consumption

The performance of implantable medical devices is limited by power consumption, and there are several approaches for recharging batteries, such as the inductive-loop approach [80–83]. Nevertheless, biotelemetry links are used to operate only when the necessary data need to be transferred. For this purpose, some transceivers are economically available (Zarlink ZL70101 transceiver) [84]. The transceiver practices two signals one for wake-up, used for transmission, and the other for low power consumption sleep. Until the transceiver obtains a wake-up signal in the 2.45 GHz ISM band, it stays in sleep mode. In regular mode, the system is completely loaded, transmits data in the MICS band, and then switches back to sleep mode after the transmission is finished. When a patient event is recognized or the doctor specifies a schedule, the external controlling device is programmed to automatically wake up the system [22].

Owing to the limitations of battery lifetime and capacity, wireless power transfer (WPT) techniques are applied in medical implants and biomedical sensors and in different biomedical applications such as pacemakers, brain stimulators, capsule endoscopes, and retinal prostheses. Wireless power transfer offers a safe and simple solution that eliminates power capacity barriers and lessens the challenges involved with surgical battery replacement. Recently many biomedical implants, including capsule endoscopes [85], heart rate sensors [86], brain simulators [87], and left ventricular assist devices [88] have been used for WPT.

**Fig. 3:**
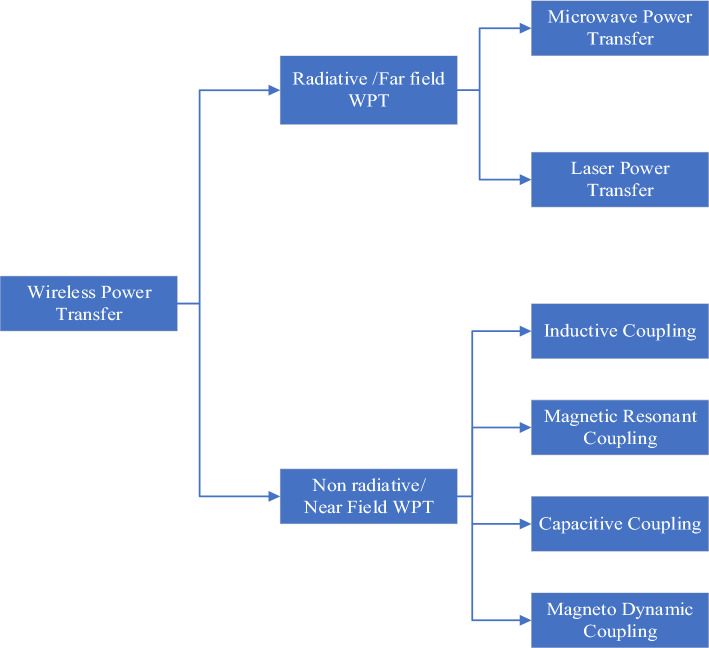
Categorization of wireless power transmission methods

On the basis of electromagnetic fields, the WPT technique can be categorized into a near-field region or nonradiative region and a far-field or radiative region [89]. The differences between radiative and nonradiative WPT are the coverage distance and operating frequency [90]. Figure 3 shows the classification of the WPT techniques. For nonradiative transmission, the transmission distance ranges from millimeters to several meters. Radiative transmission covers a large area of nearly several kilometers [91]. Nonradiative WPT can be subcategorized into various types: magnetic resonant coupling (MRC), inductive coupling (IC), capacitive coupling (CC), and magneto dynamic coupling (MDC). Far-field WPT transmission involves laser and microwave power transfer and can be transferred over hundreds of meters [92–95].

## Review of implantable antennas for leadless pacemakers

**Fig. 4:**
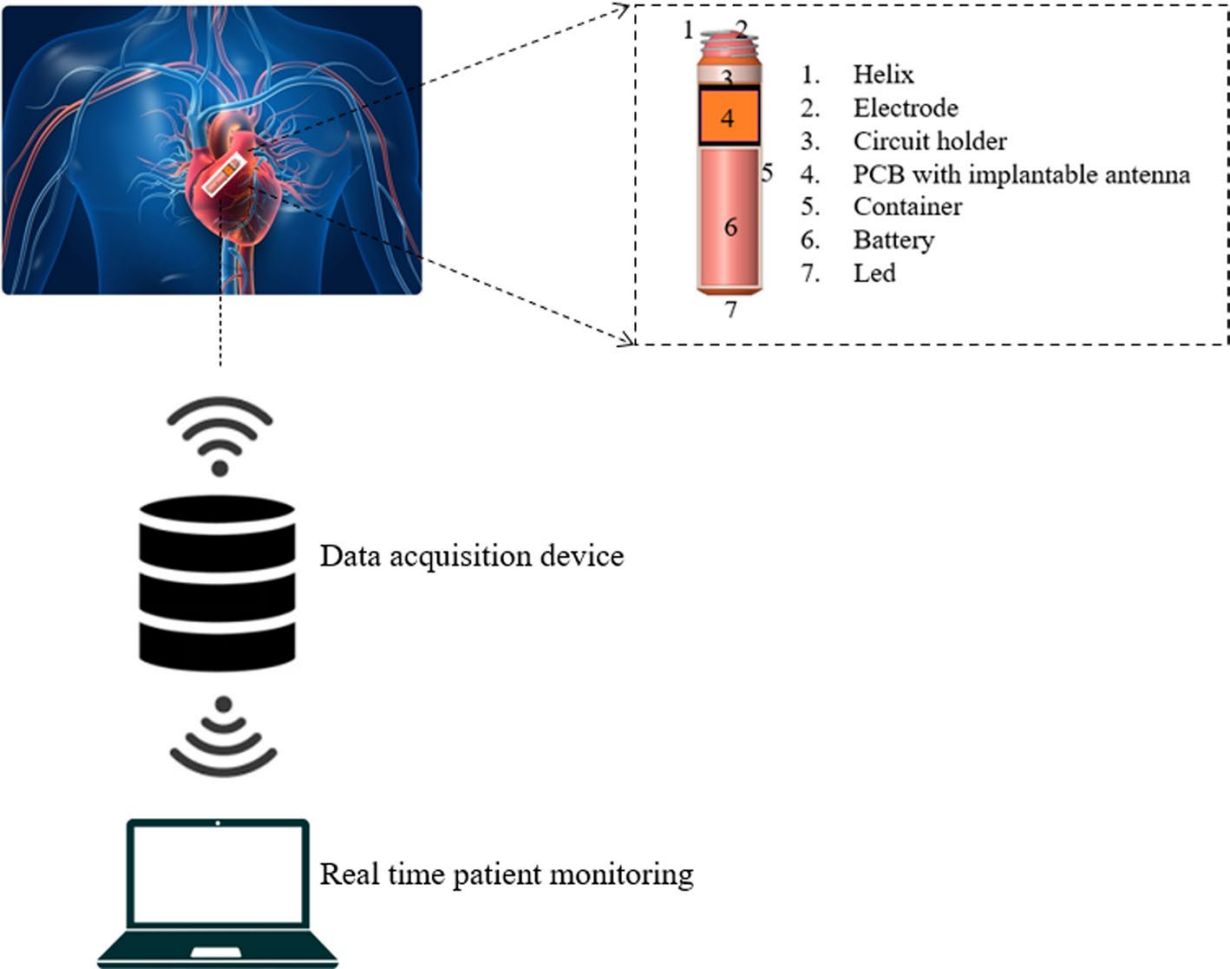
Overview of leadless pacemaker

Currently, arrhythmia-related cardiac dysfunction affects hundreds of millions of people worldwide. To encourage healthy aging and reduce the number of cardiovascular disease (CVD)-related premature deaths, affordable healthcare services are necessary. In addition, remote monitoring is used to minimize face-to-face interactions between patients, healthcare professionals, and doctors and it also lowers exposure to a variety of infections. The commercially available pacemakers are the Micra Transcatheter Pacing System (Medtronic) and the Nanostim Leadless Pacemaker (Abbott). Figure 4 shows an overview of the architecture of leadless pacemaker and its components.

### Conventional pacemaker (CP) versus leadless pacemaker (LP)

Pacemakers are medical devices that assist patients in controlling their heartbeat in the event of arrhythmias or other cardiac conditions. Leadless pacemakers and conventional pacemakers are the two primary types of pacemakers. The conventional pacemakers consist of pulse generator (battery with electric circuitry) and leads. The CP requires a surgical incision to place the device within the heart chambers. However, it has many complications such as infections, lead misplacement, lead to damage and need regular maintenance as well as battery replacement. The leadless pacemakers are very small in size, can be anchored to the heart chamber with tiny tines and are minimally invasive. The LP is lead free so it eliminates the risk of infection, fracture or lead displacement. However, LPs have some limitations such as single chamber pacing and battery life.

### Design challenges

Research on deeply implantable antennas designed for leadless pacemakers is limited due to the challenges of creating such antennas in the confined space available, while also accounting for the loss of signal caused by human tissues and the surrounding electronic modules. Pacemakers have a limited battery life, so the design of the antenna must be power-efficient to prolong the device’s operational life. Additionally, some designs incorporate energy harvesting techniques that require an efficient antenna design to capture and convert energy from external sources. The impedance of human tissue can also affect the impedance of the antenna, necessitating careful matching to ensure minimal reflection and maximum power transfer. Achieving a wideband impedance match can improve signal quality and reliability.

The two main types of leadless pacemaker antenna designs are conformal and flat. Specifically, flat antennas must be mounted on top of the biotelemetry module, which is housed inside the pacemaker capsule, or on the PCB holder. In contrast, conformal antennas must be encircled by the curved surface of the pacemaker capsule. Recent research has demonstrated the miniaturization, wideband characteristics and wireless power transfer techniques.

### Various approaches employed in the literature

The spiral-shaped antennas are used to downsize the implantable antenna footprints, as well as to improve the multiband characteristics for leadless pacemakers with biotelemetry applications at the MICS, ISM and midfield frequency bands. A miniaturized volume of $17.15 {mm}^{3}$ was achieved by using the high dielectric substrate/superstrate, spiral-shaped radiator with two symmetrical arms and open-end slots in the ground [96]. An ultra-compact implantable antenna with a volume of $4. 5 {mm}^{3}$ was achieved with a folded meander line radiator, a defective ground structure and a high dielectric substrate for wireless cardiac pacemaker application in the ISM band. A ceramic aluminum oxide film was used to coat the whole system, to maintain biocompatibility [97]. To enhance the reliability and effective telemetry link between the pacemaker and external devices the circular polarization technique is used in the pacemaker. The asymmetrical U-shaped meander line radiator with different slots in the ground without any shorting pin was used to obtain the circular polarization and $7.7 {mm}^{3}$ antenna footprint at the 915 MHz ISM band for pacemaker applications [98].

**Fig. 5:**
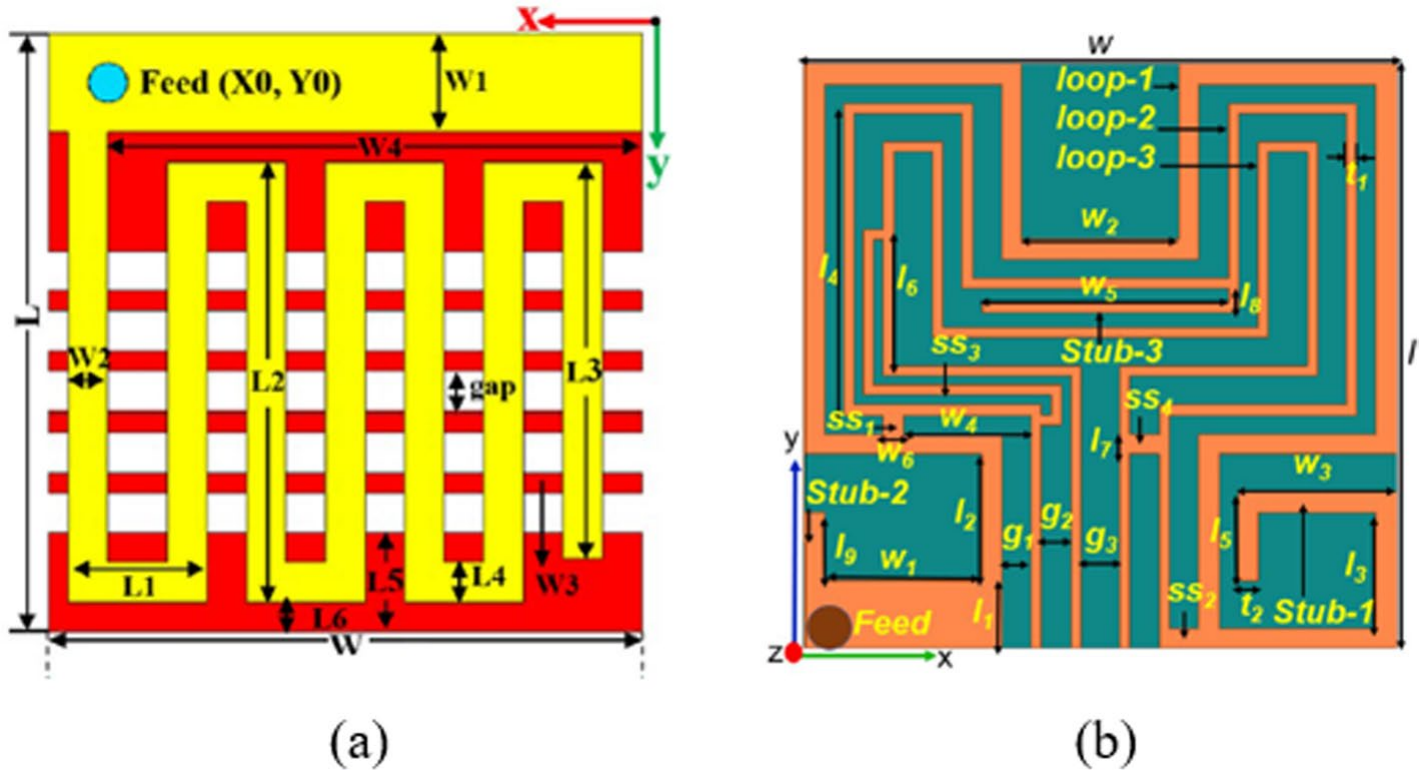
**a** Flat type meander line antenna [97]. **b** Conformal type loop antenna [101]

The ultra-wideband characteristics enable high data rate transmission between the pacemaker and external device and prevent detuning. The reported antenna has a compact size of $9.44 {mm}^{3}$ and is validated with a homogeneous heart phantom for covering the WMTS, midfield and ISM band frequencies to obtain a 3380 MHz ultra-wide bandwidth. The large rectangular slot in the radiator and the insertion of a meandering line in the ground plane are used for miniaturization and ultra-wideband characteristics [99]. The large circular, semicircular slots in the radiator and meander line in the ground are used to achieve the ultra-wide bandwidth of 3040 MHz and a miniaturized volume of $10.66 {mm}^{3}$ and it cover three different ISM band, midfield and WMTS frequency bands [100]. Figure 5 shows the flat and conformal types of antennas used in the literature.

The circularly polarized loop antenna inspired by the Hilbert curve was reported in [101]. A high dielectric constant substrate/superstrate and fractal geometry were used to attain a small footprint of $9.1 {mm}^{3}$ at the WMTS and ISM frequency bands. The designed antenna features a conformal shape that perfectly fits within the leadless pacemaker’s capsule, and is enclosed by dummy electronic components. Afterward, the integrated prototype is simulated deeply within the center of the multilayer realistic heart model. Recently, the multiple input multiple output (MIMO) implantable antennas have been developed to increase the spectral efficiency and data rate in pacemaker applications. Compared to single antenna systems MIMO communication systems provide high capacity and low-loss transmission/reception without the need for additional power. The dual-band circularly polarized triple band MIMO antenna was presented in [102]. By utilizing ring slots, rectangular slits, shorting pins, and a meandering structure, the developed antenna is able to reach compact dimensions of $32.3 {mm}^{3}$ and accomplish CP properties at the WMTS, ISM and midfield frequency bands. The Hilbert curve-based fractal MIMO antenna was presented in [103], in which the fractals are used to improve the current path acquire the multiband characteristics and downsize the antenna geometry to $5.69 {mm}^{3}$. Leadless pacing devices are equipped with an ultra-compact MIMO antenna that allows low-loss MIMO transmission to an external MIMO antenna for remote cardiac monitoring on a 5G IoT platform.

Pacemakers and other implanted medical devices face significant challenges in terms of is battery lifetime. To extend its service life, which could result in patients requiring additional surgery, the battery must be changed. It is necessary to create an implantable antenna that can transmit power and facilitate communication to prolong battery life and reduce the risk of the patient’s life. The emergence of wireless power transfer (WPT) technology offers potential remedies for this issue. The split resonant ring-based implantable antenna is used to obtain the ultra-wideband characteristics has a footprint of $91.44 {mm}^{3}$ at the ISM band [104]. The silicone head is used for the biocompatibility purposes. Furthermore, a novel wireless power transfer (WPT) system is developed by including a tiny metasurface to improve WPT efficiency and prolong the lifespan of implantable devices. The loop-based patch and meander line in the ground are used to attain the minuscule dimensions of $10.6 {mm}^{3}$ and ultra-wide bandwidth. The proposed antenna was merged with a multisection rectifier with wideband capabilities to convert the received radio frequency (RF) energy into direct current (DC). The rectifier’s RF-dc conversion efficiency has been increased by the use of spatial power combining techniques [105]. These methods involve the independent generation of two signal components, 1.5 and 1.52 GHz, which are then transmitted via an ultra-wideband horn antenna. Table 2 lists the various types of antennas and their parameters used in previous studies.

**Table 2:** Implantable antenna for leadless pacemakers

Ref no.	Antenna type	Dimension of antenna (mm^3^)	Frequency of operation (MHz)	Bandwidth (MHz)	Gain (dBi)	Substrate/superstrate used
96	Spiral–shaped	$7\times 6.5\times 0.377$	402, 1600, 2450	148, 171, 219	− 30.5, − 22.6, − 18.2	Rogers RT/Duroid 6010
97	Meander line	$3\times 3\times 0.5$	2450	22	− 24.9	Rogers 3010
98	Meander line	$5.2\times 5.6\times 0.25$	915	170	− 23	Rogers RO3010
99	Patch with slots	$6\times 6.2\times 0.254$	1400	3380	− 28.9	Rogers RT/Duroid 6202
100	Patch with slots	$6\times 7\times 0.254$	1400	3040	− 20.02	Rogers RT/Duroid 6202
101	Loop	$6\times 6\times 0.254$	1400, 2450	17.1, 12.8	− 32.7, − 25.92	Rogers RT/Duroid 6010 LM
102	MIMO	$\pi \times {\left(4.5\right)}^{2}\times 0.508$	610, 1650, 2450	200, 200, 320	− 41.7, − 38.4, − 28.9	Rogers RO6010
103	MIMO	$3.4\times 6.6\times 0.254$	2400, 5800	44, 40	− 31.6, − 21.4	Rogers 6010LM
104	Split resonant ring	$12\times 12\times 0.635$	402, 920	1232	− 32, − 34	Rogers 6010
105	Patch with slots	$6\times 7\times 0.127$	915, 1400, 2450	3040	− 25.73, − 18.56, − 15.33	Rogers RT/Duroid 6002

## Review of implantable antennas for capsule endoscopy

The capsule endoscopy system is a noninvasive method which a tiny, ingestible capsule embedded with a wireless transceiver system and camera used to obtain images of gastrointestinal tracks for disease diagnosis.

### Conventional endoscopy versus capsule endoscopy

Conventional endoscopy is ideal for therapeutic interventions and high-resolution visualization of the upper and lower gastrointestinal tracts. Although it is intrusive and anesthesia is necessary, it provides direct control and the opportunity to provide therapies as necessary. For a thorough, noninvasive evaluation of the small intestine and entire GI tract, wireless capsule endoscopy is the best opportunity. Although it is safer and more pleasant for patients, its usage is restricted to diagnosis and does not have any therapeutic potential.

### Design challenges

**Fig. 6:**
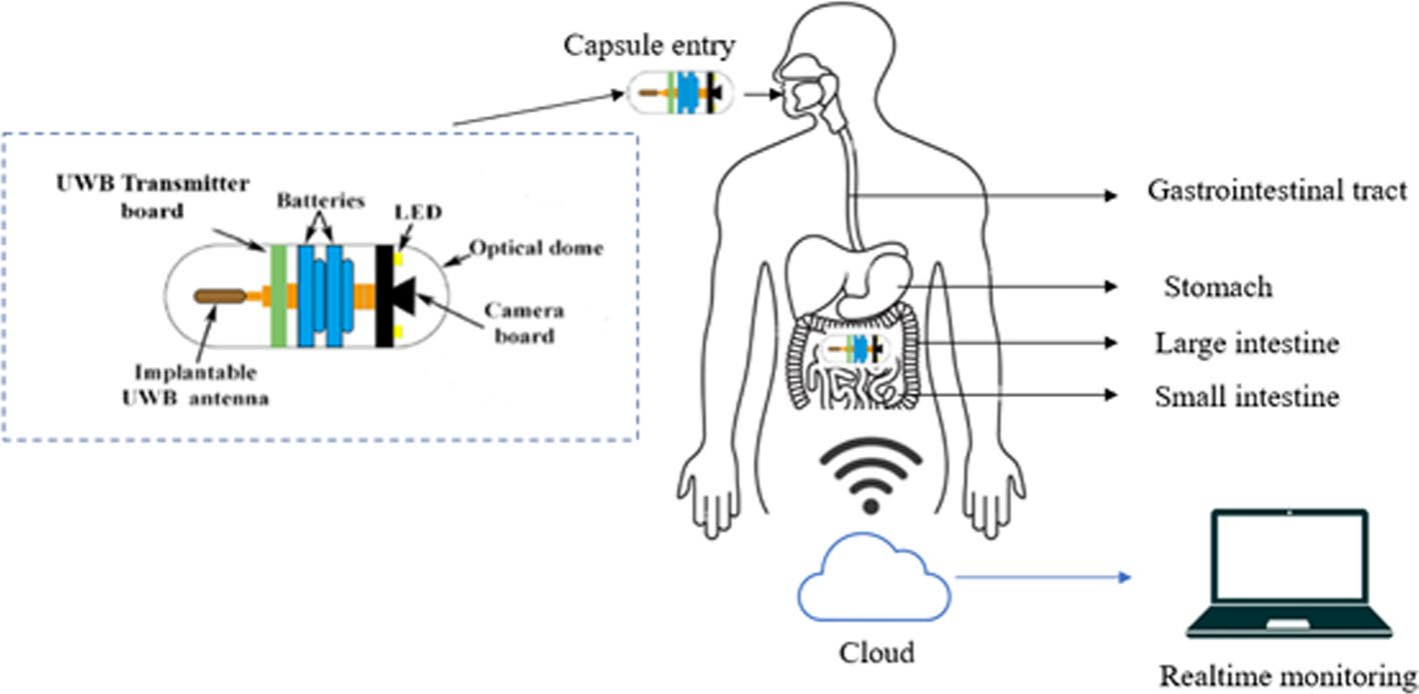
Overview of the capsule endoscopy system

To determine this parameter the capsule antenna should be small in size and should be able to move through various body parts. Within the capsule’s constrained space, the antenna must perfectly harmonize with other parts including the camera, battery, and transmitter. For high data transmission rates, ultra-wideband capsule antennas are needed. For extended periods of time in contact with human tissues, the antenna materials must be safe and biocompatible. Avoiding any harmful or reactive materials is part of this. It is necessary to encase the antenna and other electronic components in a biocompatible material that shields them from physiological fluids while enabling signal transmission. There is a slight chance that the capsule will become lodged in a constricted or narrowed section of the GI system and need to be surgically removed. Therefore, the antenna for capsule endoscopy requires a small size, wide bandwidth, and omnidirectional radiation pattern to achieve successful data transmission from the capsule endoscope to the digestive system. Figure 6 shows the detailed operation of wireless capsule endoscopy.

### Various approaches employed in the literature

A flexible magnetic-based spiral antenna ($\pi \times {(8)}^{2}\times 26 {mm}^{3}$) with a conformal structure and a dielectric-based meandering line antenna ($\pi \times {(6)}^{2}\times 0.64 {mm}^{3}$) with a planar structure were proposed for use in body applications [106]. Similarly, on-body applications for MICS band spiral antennas ($20 \times 6.8\times 1.27 {mm}^{3}$) and ISM band meander line ($42 \times 10\times 1.6 {mm}^{3}$) were also designed. Both the spiral structure and meander line are used for miniaturization. To decrease the multipath interference and increase the bit error rate circularly polarized antennas are proposed. A dual-band circularly polarized and ultra-miniaturized antenna was presented [107]. The circular polarization and miniaturization are obtained by the meander line structure combined with slots and a defective ground structure without any vias provides the smallest footprint of $2.11 {mm}^{3}$ in the MICS and ISM bands. The proposed structure is formed like a capsule with a copper cylinder and copper strips used to enclose the capsule to provide more space to other components [108]. The omnidirectional and wide bandwidth was achieved with the help of copper strips. The polyimide shell was used to encapsulate the antenna system to provide biocompatibility and reduce the electromagnetic interference produced by the surrounding tissues. The dimensions of the proposed cylindrical structure are $20\times 11\times 0.07 {mm}^{3}$ operates for the WMTS and ISM bands.

An ultra-thin and ultra-miniaturized antenna was introduced in [109] in the ISM band for deep tissue applications such as capsule endoscopy. Miniaturization was achieved by placing slots in the radiator and ground as well as the thin substrate/superstrate material Rogers ULTRALAM with 0.2 mm thickness, and the volume of the proposed antenna was $7.8 {mm}^{3}$. The dual band feature is an important aspect of an implantable antenna. This feature enables simultaneous data transmission and power transfer at two distinct frequency bands. The dual band and miniaturized volume of $42.69 {mm}^{3}$ were presented in [110]. The dual band and miniaturization were acquired by using the shorting pin, open-ended slots, semicircular slots and middle slot in the patch. A biotelemetry link was created to validate reliable transmission. The presented implantable antenna can be used in conformal or planar forms [111]. To make the conformal structure the antenna was mounted in the outer wall of the capsule. The meander line structure was used to achieve a miniaturized volume of $2.5 {mm}^{3}$, and 0.2 mm thickness of polyethylene material was used for biocompatibility. The conformal dual polarized ultra-wideband antenna was designed in [112] for wireless capsule endoscopy applications. The dual polarization was attained by irregular rectangular loop, with a step shaped monopole, these two loops are separately printed on top and bottom of the dielectric substrate. The slot-based patch conformal antenna was embedded into the $17.7\times 8.9 mm^{2}$ capsule encircled with flexible polyimide material for tissue independent ingestible and implantable capsule applications in Med radio band [113]. The proposed antenna achieves the ultra-robust impedance characteristics. The conformal low-profile microstrip antenna was introduced for ingestible and implantable capsule endoscopy as well as animal biotelemetry applications. The hybrid numerical analytical approach was used for a miniaturized size of $7 mm\times 17 mm \times 50 \mu m$ at 433 MHz [114].

### MIMO implantable antenna for capsule endoscopy

For capsule endoscopy applications, MIMO antennas provide several benefits over single-element antennas, including expanded coverage, higher data rates, better signal quality, multiplexing of spatial signal features without any additional power, and interference resilience, improving diagnostic capabilities. But the major limitation of MIMO antenna with small geometry is isolation. The proposed conformal two-port MIMO antenna with meandered loop structure has a footprint of $5.928 {mm}^{3}$, and it covers MICS, WMTS and ISM band of frequencies [115]. The proposed wireless capsule endoscopy consists of MIMO antenna, two camera modules, batteries, LED and PCB with dummy electronics. The polyimide material is used for biocompatibility purpose and transparent mica is used for encapsulate the capsule lid to enhance the image quality. The low-profile MIMO covers MICS and ISM band for capsule endoscopy and scalp implantation. The miniaturized dimensions of $13.03 {mm}^{3}$, was achieved by using shorting pins, rectangular and arc shape slots on the patch and open-ended slots in the ground [116]. The capsule was validated on both planar and conformal form. To verify the effective communication link budget analysis was made.

The miniscule, high data rate and two element MIMO antenna was presented [117] for capsule endoscopy application at ISM band. The miniaturized size of $2.52 {mm}^{3}$ was achieved by using high-permittivity substrate, defective ground and meandered loop radiator. The slots and I shape stubs are used to provide high isolation between elements. The four-port MIMO antenna was projected in [118] for wireless endoscopy application at ISM band. The MIMO antenna contains four probe fed meandered loop with common ground plane with 0.5 mm edge to edge separation has a dimension of $4.09 {mm}^{3}$. The compact two-port dual-band implantable antenna was proposed in [119]. The compactness of $15.3 {mm}^{3}$ was achieved by using the meandering slots and ground plane slots without shorting pin. Additionally, no mutual coupling reduction strategy was used to improve port isolation. Rather, the meandering lines were purposefully crafted to offset the field effects that impede currents flowing in opposite directions on nearby lines within the radiating patch, which transpire at both resonances. The novel approach of SISO water-based implantable antenna (WBIA), which can be quickly transformed into a MIMO antenna for implantable device [120]. Because of the water's extremely high permittivity, the WBIAs’ size was greatly decreased. The volume of $315 {mm}^{3}$ MIMO antenna downsizing is possible with high-permittivity water, the capacitive effect of radiator and water-based ground plane at ISM band. Table 3 lists various antennas used for the capsule endoscopy application in literature.

**Table 3:** Implantable antenna for capsule endoscopy

Ref no.	Antenna type	Dimension of antenna (mm^3^)	Frequency of operation (MHz)	Bandwidth (MHz)	Gain (dBi)	Substrate/superstrate used
106	Planar and conformal combined	$\pi \times {8}^{2}\times 26$	402, 1500, 2450	23, 292, 67	− 35.63, − 32.32, − 31.43	Magnetic flexible substrate, FR–4
107	Patch with slots	$6.5\times 6.5\times 0.05$	915, 2450	123.5, 154.4	− 28.2, − 24.5	ULTRALAM 3850HT
108	Circular	$11\times 22\times 1.524$	732–1699	967	− 28.2, − 29.4	Rogers 4350B
109	Patch with slots	$6\times 6.5\times 0.2$	2450	480	− 16.5	Rogers ULTRALAM
110	Semicircular slots	$8.2\times 8.2\times 0.635$	1400, 2450	240, 400	− 29.4, − 30.4	Rogers RO3010
111	Meander line	$12\times 6\times 0.127$	402, 433, 915, 2450	3330	− 32.5, − 30.4, − 17.9, − 19.0	Kapton
112	Patch with slots	$21.7\times 14.8\times 0.254$	4000	13180, 12850	− 23.8, − 18.5	Rogers 6010
114	Flexible microstrip	$7 \times 17 \times 0.05$	434	Not specified	− 22	Rogers ULTRALAM 3850HT
115	MIMO–meander loop	$15.5\times 4.5\times 0.085$	402, 433, 915, 1400, 2450	2200	− 41.41, − 39.89, − 33.10, − 37.30, − 34.74	Polyimide
116	MIMO–semicircular slots	$\pi \times {5.65}^{2}\times 0.13$	433	145	− 30	Rogers RO3010
117	MIMO–meander	$5\times 4.2\times 0.12$	2450	620	− 20.6	Rogers RO3010
118	MIMO–meander	$6.2\times 5.2\times 0.127$	2450	350, 350 470, 120	− 20.1	Rogers RO3010
119	MIMO–meander	$10.8\times 5.6\times 0.254$	915, 2450	148.5, 89	− 32.15, − 22.2	Rogers RO6010
120	MIMO–circular	$\pi \times {5}^{2}\times 4$	2450	410	− 19.3	Water enclosed with bio-plastic material polylactic acid

## Review of implantable antennas for intracranial pressure monitoring

It is difficult to use clinical measurements of intracranial pressure (ICP) to predict head wounds and ailments of the brain and stroke to prevent death and disability.

### Traditional methods versus implantable antennas for intracranial pressure monitoring

The intraventricular catheter (ventriculostomy), subdural/epidural/parenchymal sensors, methods are used to measure intracranial pressure through invasive methods, and transcranial Doppler, and magnetic resonance (MR) methods are used to measure the ICP by imaging methods. In the above methods the catheter needs to be inserted into the brain nerves and the cerebrospinal fluid value needs to be read via wired methods. Using the implantable antenna, a wireless sensor system implanted in the cranial cavity measures the ICP and transmits data to an external receiver via an implantable antenna. While traditional methods of ICP monitoring are highly accurate and widely used, they have significant risks and limitations, particularly with respect to invasiveness and patient comfort. Implantable antennas offer a promising alternative, providing minimally invasive, continuous, and wireless monitoring. However, they also present new challenges, particularly in terms of miniaturization, biocompatibility, signal transmission from complex brain environments and power management.

### Design challenges

The in-body antenna design was complicated because of the unpredictable nature of human tissue and organs, and it absorbs most of the antenna radiation. The important considerations when designing implantable antennas for ICP monitoring are their wide bandwidth, broad radiation pattern, biocompatibility and good signal transmission capability.

### Various approaches employed in the literature

Previously ICP measurements under megahertz frequencies required large inductive links, after which they were moved in terms of microwave frequencies with the help of a piezoresistive sensor, oscillator, and chip antenna [121]. The planar inverted F antenna was used to monitor the ICP in the ISM band and monitoring with a scalp phantom matching the implant environment was implemented to estimate the operating time, frequency, emitted temperature, and radiation of the antenna to imitate its physiological surroundings [40].

**Fig. 7:**
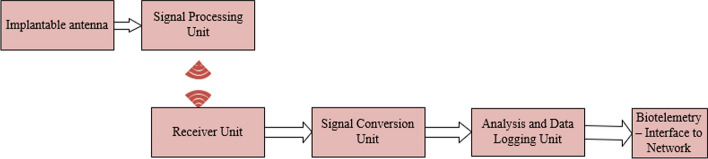
Block diagram of the wireless intracranial pressure monitoring system

Figure 7 shows the block diagram of an intracranial pressure monitoring system; it has an implantable antenna that is used to measure the intracranial pressure and then the vital body parameter is processed by a signal processing unit after it is transmitted through the wireless medium. The receiver unit consists of a signal conversion unit, analysis unit, and data logging unit and finally, it is interfaced with a network for biotelemetry purposes.

The employment of a tiny ($6 \times 5 mm^{2}$) coplanar implant antenna with a sensor stage to transmit the recorded intracranial pressure to the skull and generate a link budget to transport the data in the 0.5–1 m range is part of the minimally invasive intracranial pressure monitoring technique [122]. The intracranial pressure is monitored at 2.45 GHz via annular slot antennas, which have a better radiating efficiency than previously utilized strip antennas, such as the printed inverted F antenna. The performance deviation can be controlled by the shielding layer to the outer wall of the cable in a vector network analyzer test and an appropriate grounding technique is used for the cable exterior conductor and the inner feed point [123]. On the basis of the temperature distribution the ICP is also monitored with a dual-band implantable antenna. The spherical shape human head model is used for numerical analysis. A temperature range from $0.5-2.7^\circ{\rm C}$ is generated close to the implantable antenna on the basis of the IEEE basic exposure limitations [124]. The loop antennas are used for wireless ICP monitoring. In this loop, the antenna can penetrate a maximum distance of 19 mm to the skin which is 8 mm greater than that in earlier models, and it initiates penetration of the implant 11 mm into the skin [125].

An ultra-miniaturized dual-band implantable antenna system was developed and the projected system was integrated with microelectronic elements, batteries, and a supple implantable antenna. Link budget calculations are used to verify the wireless communication ability and establish the transmission range from human tissue to the outside world. Ultra-miniaturization is also achieved by using a spiral radiator, a shorting pin, and a ground plane with several slots. The current path is extended by using spiral geometry and shorting pins. The gain is increased by using the slots in the ground plane and moving the resonance frequency to lower bands. The superstrate acts as an insulating layer, to prevent radiation from occurring in the outside environment [126–129]. To reduce the design complexity, some implantable antennas are made with a slot less and via less ground planes. The biotelemetry link is tested with link margin calculations and the communication link can be launched at a distance of 4.5 m [130].

The vertical end-fire antenna with a Vivaldi shape operates at 3–5 GHz for high-speed air communication to the brain–machine cooperative application. To prevent radiation distortion and avoid gain loss due to the lossy tissue environment, the designed Vivaldi antenna is rooted vertically at the altitude of the skull. Miniaturization is achieved by using slot arrays which are also used to increase the gain and bandwidth [131]. Deionized water with high permittivity is utilized as an insulator to accomplish miniaturization and deionized water-based padding is employed to separate the antenna to improve wideband properties and impedance matching. To achieve the broadside frequency response, the proposed antenna consists of a tapering treaded microstrip feed line and an enhanced rectangular slit etched on the ground plane [132]. The dual-band four-port self-quadruplexing implantable antenna was introduced for scalp implantable applications [133]. A miniaturized size of $22.62 {mm}^{3}$ was achieved by using semicircular slots, a semicircular meandered radiator and high dielectric substrates at three ISM bands and WMTS band. Furthermore, three SDRs are used to verify this antenna's simultaneous transmit and receive (STAR) capability.

In Table 4, the implantable antennas designed by different investigators for intracranial pressure monitoring are consolidated. It contains the antenna type, operating frequency, and antenna parameters. Mostly the meandered line structure and patch antennas are used at the WMTS, and ISM, and some ultra-wide bands are also used for intracranial pressure monitoring applications. Typically, high dielectric constant substrates are used to achieve miniaturization and improve the gain and overall implantable antenna performance.

**Table 4:** Implantable antennas for intracranial pressure monitoring

Ref. no.	Type of antenna	Dimensions of antenna (mm^3^)	Frequency of operation (MHz)	Gain (dBi)	Bandwidth (MHz)	Substrate/superstrate used
40	Planar inverted F-antenna	$6\times 6 \times 0.787$	2450	− 24.8	Not specified	FR4/silicon
122	Coplanar implant antenna	$6\times 5\times 0.3$	2450	− 23	280	Flexible polyimide
123	Annular slot antenna (ASA)	$19.6\times 19.6\times 0.762$	2450	− 18.4	Not specified	Rogers 4003 C/silicon
124	Planar inverted F-structure	Radius – 11.9 Thickness: Not mentioned	402, 2400	− 37.89, − 13.81	70.1, 133.7	Not specified
125	Loop antenna	Not specified	2450	− 11.2	Not specified	FR4
126	Ultra-miniaturized flexible antenna	$7\times 7\times 0.2$	915, 2450	− 28.04 − 23.01	107.5, 560	Rogers ULTRALAM
127	Patch with slots	$8\times 6\times 0.5$	915, 2450	− 28.5, − 22.8	90, 210	Rogers 6010
128	Two-turn loop antenna	$2\times 2 \times 2$	300–3000	Not Specified	Not Specified	Rogers RT/Duroid 5880
129	Spiral antenna	$6\times 6\times 0.5$	915, 1450	− 32.8, − 24.8	123, 87	Rogers 6010
130	Arc-slotted circular shaped antenna	Inner radius: 2.755 Outer radius: 3.772 Thickness: Not mentioned	1320, 2440	− 24.5 20.6	80, 100	PEC
131	End fire antenna	$12\times 7\times 0.5$	3000–5000	− 15.7	2000	Taconic RF-35
132	Rectangular slot patch antenna	$10\times 9\times 0.7$	3000–14000	− 19	Not specified	Taconic TRF–43
133	Semicircular meandered resonator	$12\times 14.5\times 0.13$	168, 433, 915, 1400	− 36.24, − 34.78, − 31.39, − 30.10	36.65, 25.28, 21.22, 18.45	Rogers RO3010

## Review of implantable antennas for retinal prostheses

Retinal prostheses are reconstructive devices used for limited visualization in blind people suffering from retina pigmentosa (RP) and age-related macular degeneration (AMD), where some percentages of the retinal film is depreciated triggering impaired vision. This section elaborates on some of the retinal prosthesis implantable antennas designed by researchers.

### Design challenges

Miniaturization of retinal prosthesis implants is challenging because the designed implant needs to fit in the intraocular region to activate the intact posterior visual pathway. Similarly, biocompatibility is another criterion. Additionally, heat generation around biological tissues needs to be maintained at minimum levels.

### Various approaches employed in the literature

The intraocular element in a retinal prosthesis is designed by using the compact planar meander line dipole. This confirmed that the meander dipole can perform better than a traditional microstrip patch antenna for a retinal prosthesis [134]. The 2D and 3D pleated dipole antennas are cast off for the bioinformation telemetry link to the retinal prosthesis. While two-dimensional models have a smaller planar footprint than three-dimensional models, three-dimensional models nevertheless have better antenna properties such as gain and bandwidth [135]. A microstrip patch antenna with an RF MEMS capacitor was incorporated at microwave frequencies for both intraocular and extraocular applications. To achieve the desired resonant frequency RF capacitors are placed on the microstrip stubs. Nevertheless, the main limitation of this system is that the antenna is filled with a radial stub, so many capacitors are not loaded with stubs [136].

The microstrip-based PIFA antenna in the MICS band is suitable for increasing radiation efficiency in the inner eyeball [137]. The miniaturized circularly polarized and conformal microstrip patch antenna was designed at 2.4–2.48 GHz for retinal prosthesis application and data transfer from the prosthesis to the exterior world. Specific absorption rate distributions in the inner head phantom model at the MICS band (402–405 MHz) and two ISM band (902–928 MHz and 2.4–2.48 GHz). The MICS band and ISM band (902–928 MHz) SAR values are acceptable for the retinal prostheses, but for the 2.4–2.48 GHz band the SAR value is greater and losses are also greater than the remaining two frequency bands [138]. Modified Hilbert transform and serpentine geometries are used to achieve miniaturization and circular polarization. Both the substrate and the superstrate are made up of polydimethylsiloxane for better gain and biocompatibility [139].

The biocompatible microstrip patch antenna employed for retinal prosthesis application in the Med radio frequency band. The antenna is implemented with three dissimilar biocompatible materials namely a Silastic MDX4-4210 medical-grade elastomer, zirconia, and PEEK, and the performance is analyzed. The desired antenna tuning properties are achieved via an automated quasi-Newton method [140]. Triangularly shaped microstrip patch antennas for intraocular and planar inverted F-antennas have been investigated for extraocular purposes at 1.45 and 2.45 GHz [141]. Wire patch antennas were also used at the ISM and UWB bands for retinal prosthesis applications on the basis of Hansen and Collin’s physical boundaries. The antenna performance is evaluated exclusively by diverse eye specter counting with an eye specter examination model established similarly to that used for prototypical SMCM eyes [142]. The proposed compact rectenna system consists of a rectifier and an implantable receiver antenna and transmitter at 915 and 2450 MHz. The presented antenna is shaped by adding a semicircular ring-loaded rectangular stub and two open-ended circular annular rings to a traditional circular patch. To obtain the desired frequency bands, a shorting pin is also utilized at the patch edge. Additionally, the circular ground plane is altered by creating symmetrical slots across the four quadrants and edges to make the structure electrically compact and fit extremely dense electrodes. The volume of the proposed antenna is $0.35 {mm}^{3}$ at triple frequencies of 1.25 GHz, 2.45 GHz and 3.32 GHz [143]. Table 5 shows a few of the implantable antennas used for retinal prosthesis applications. In retinal prosthesis applications mostly microstrip patch antennas are used at Med radio and ISM band frequencies.

**Table 5:** Implantable antennas for retinal prostheses

Ref no.	Type of antenna	Dimensions of antenna (mm^3^)	Frequency of operation (MHz)	Gain (dBi)	Bandwidth (MHz)	Substrate/superstrate used
134	Planar meander line dipole antenna	$6 \times 6 \times 1.5$	1410	1.7, 5.16	20	Si
135	Folded spiral antenna-2D, 3D, 3D rotational	$5.25\times 5.25\times 0.4$ $5.25\times 5\times 4$ $5.25\times 5\times 4$	3600	4.9, 1.4, 5.4	0.88, 1.3, 0.97	Copper
136	Microstrip patch antenna	$6 \times 6$ $25 \times 25$	1400, 2350	Not Specified	Not Specified	Not specified
137	Microstrip PIFA antenna	$12 \times 12$	402–405	− 24.31	39	Rogers RO3210
138	Microstrip patch antenna	$18 \times 22 \times 9.5$ $19.6 \times 29.4 \times 10$ $9.8 \times 14.7\times 7.5$	402, 915, 245	3.28, 3.06, 4.13	0.3, 4.575, 12.25	Alumina/Rogers RO3210, Mica
139	CP microstrip patch antenna	$5.8 \times 6.5 \times 2$	2450	− 50	200	Polydimethylsiloxane
140	Circular shape planar inverted F-antenna	${R}_{g}\ge 6.02$ ${R}_{p}\ge 6$	406	− 36.64, − 36.82, − 41.02	13.95, 13.63, 13.51	PEEK Silastic MDX-4210, Zirconia
141	Microstrip patch antenna, PIFA	$7\times 6.93 \times 0.63$ $28\times 28 \times 1.43$	1450, 2450	− 35, − 36	900, 60	Rogers RT Duroid 6010
142	Wire patch antenna	$3\times 3 \times 0.64$	2450, 5800, 8000	− 18.4 − 3.5 − 4.7	9000	Flexible polyimide
143	Circular shape with slot	$6\times 6\times 0.05$	1250, 2450, 3320	− 38.2, − 30.7, − 20.5	330, 420, 410	Polyimide

## Review of implantable antennas for bone implants

Bone is a very important part of the body; it provides mechanical support to tissues and organs and is also used to store minerals. Osteoporosis occurs due to a deficiency of vitamin D, which leads to bone brittleness and bone cracks. An accident is also typically the basis of cracks or defects in the bones. Bone implants refer to artificial devices made of materials such as titanium, stainless steel, or ceramic that are surgically placed into bones to replace missing or damaged sections. They can be used to treat a variety of conditions, including fractures, osteoporosis, and joint replacements. The goal of bone implants is to restore the structure and function of the affected bones and to promote healing and recovery.

### Traditional monitoring versus implantable antenna for bone fracture monitoring

X-ray, CT, magnetic resonance imaging, ultrasound and physical examination methods are used to identify bone fractures. The traditional methods involve radiation exposure and only a limited area is covered. The physical examination provides functional information, but lacks interior visualization. Real-time monitoring and early detection are possible using the implantable antenna.

### Design challenges

The bone implant dielectric material should be biocompatible and durable. The attenuation must be reduced and the signal transmission must be increased. The bone implant size should be too small be fixed within the minimum space. Therefore, the cutting-edge antenna is designed to maximize performance in the body's intricate environment.

### Various approaches employed in the literature

The flexible loop antenna was cast off at the MICS and ISM bands for biomedical bone implant applications. The magnetic type antennas provide improved attainment inside the human body which is nonmagnetic, so loop-type structures are used. The meander-type radiators are used to provide a larger current path to shift the resonant frequency and provide the anticipated Med radio frequency band [144].

**Fig. 8:**
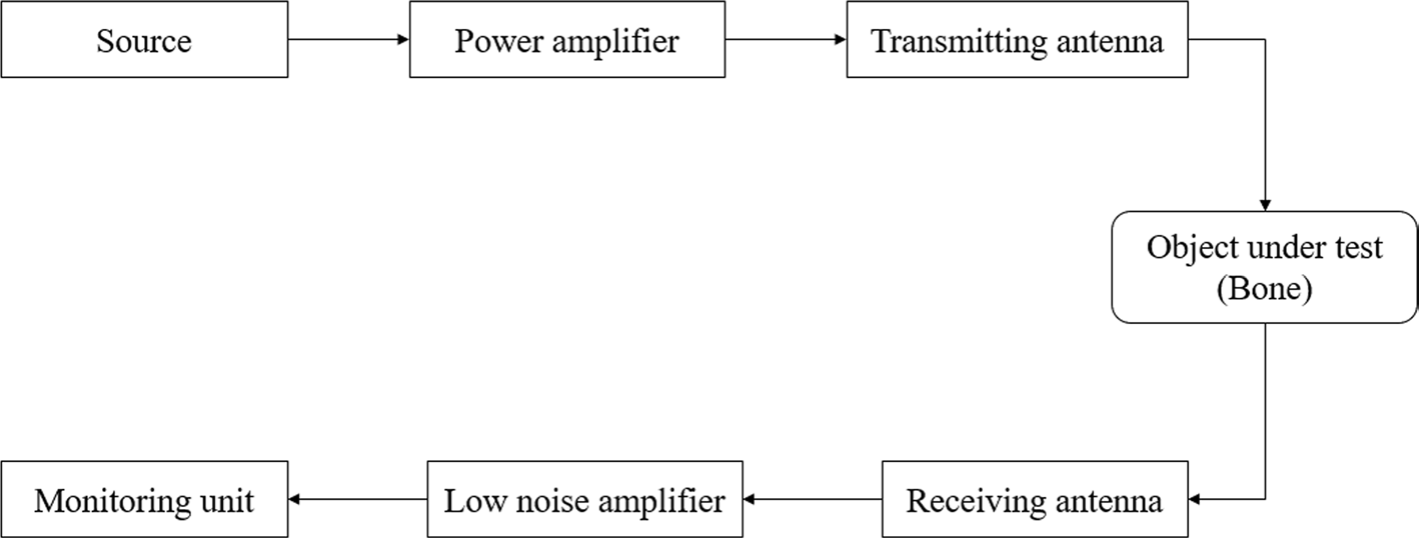
Schematic architecture of the microwave system for bone health analysis

Figure 8 is a schematic of the use of a microwave system for bone strength analysis. The microwave signal is generated by the source and amplified via the power amplifier. It is passed through the transmitter antenna and bone part under test and received by the receiver antenna. After that, it is applied to the low-noise amplifier to remove the noise. Finally, it is applied to the processor to access the output

Furthermore, an antenna structure containing dual monopoles and a metallic plate was used to monitor the healing of the bone fracture. The magnitude of S_21_ describes the fracture between the bones that are restored progressively by changing from the blood to the bone marrow and bone cortex [145]. The developed spiral-type loop antenna, is placed on the tibial bone. The aperture-type structure is modeled between the two complementary spiral layers to achieve end-fire single-sided radiation [146]. The UHF antennas are required to noninvasively monitor bone fracture at 860–960 MHz. The two matching antennas are located on the front and back adjacent to the fractured bones and based on the S—parameters difference the position of the crack can be identified [147]. Helically shaped implantable antennas are used to detect bone fractures without any contact with the bone. The fracture bone characteristics are examined through changes in the dimensions of the biological material. When the fracture width changes the dielectric properties of the antenna also change, so the fracture location can be easily identified [148].

A dual UWB stubbed monopole antenna is designed to record bone mineral density in a noninvasive manner at 3–7 GHz. Osteoporosis can be characterized by bone mineral density. A microstrip line, a modified ground plane, and a circular radiating route with a slot make up the design of a rounded stubbed monopole antenna. The ground plane is square, and on top of it, a square stub is combined to increase the bandwidth [149]. For bone implants at the 402–406 MHz Med radio band and 433–434 MHz ISM band, a patch antenna with spiral split rings is utilized with a biotelemetry link at a 12-m distance indoors. The antenna model was verified in both adult and child legs [150]. At 2.45 GHz, a small narrowband monopole antenna is used for primary-level bone crack diagnostics. The antenna design consists of a rectangular ground plane and an enhanced hexagon-shaped radiator with six integrated triangle slits on the bottom edge. The antenna simulation results are validated with pig bones and tissues [151].

**Fig. 9:**
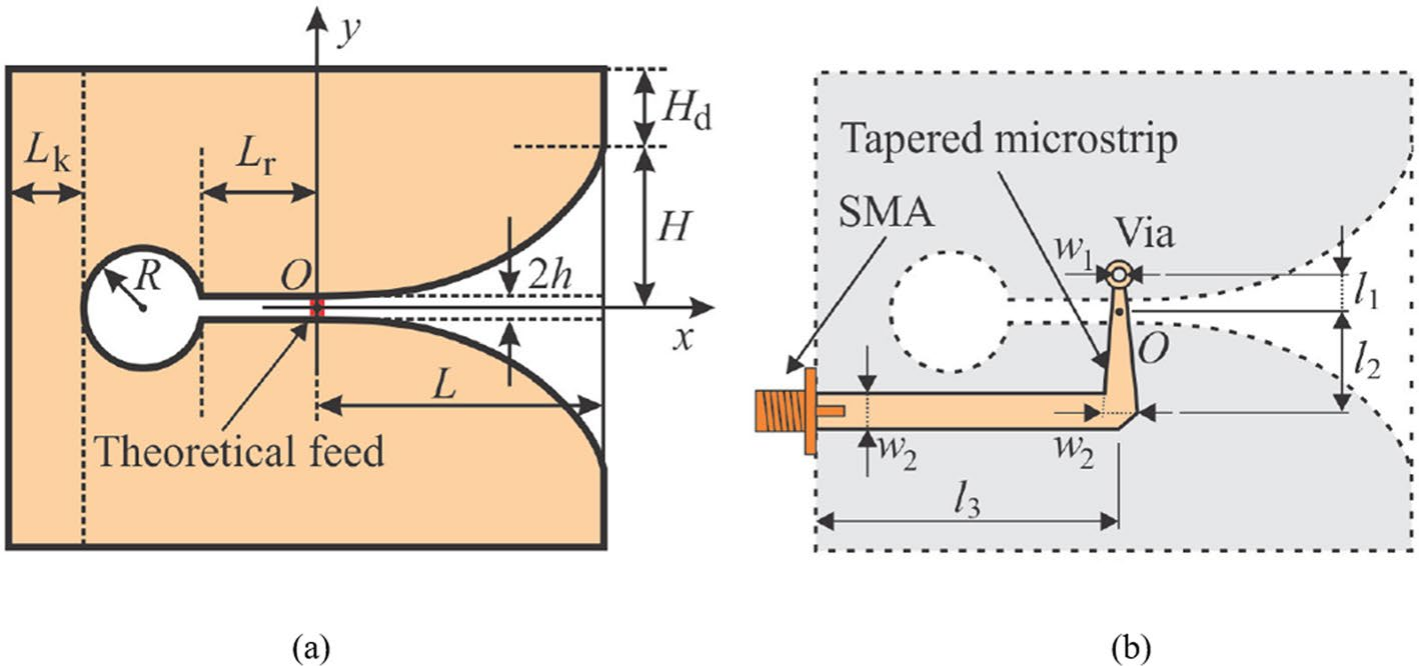
Implantable antenna for orthopedic applications: **a** top view, **b** bottom view [155]

The proposed method involves implanting a half-wave dipole antenna with a length of 116 mm in the fractured arm and measuring the transmitted power and reflection coefficient at the antenna's far-field exterior of the body at 402 MHz [152]. An additional layer is added to the humerus to mimic fracture, which causes a rise in the loss of the electromagnetic field in human tissue and a subsequent decrease in the transmitted power. When fractures occur, the average transmitted power density (APDs) varies from 11.54 to 15.75% depending on the fracture type. Additionally, the reflection coefficients increased from − 22.35 dB to − 22.65 dB compared with those of normal bone, indicating a change in the bone healing status. A conformal printed antenna that is deeply implanted at 2.45 GHz is shown. A coaxial-cable-fed trapezoidal radiator that is intended to transmit biological signals gathered by appropriate biosensors within the body using the hip implant as its ground plane. The system, which included a conformal radiator, a biocompatible gypsum-based dielectric, and a metallic (or comparable) hip implant, was evaluated by submerging the 3D-printed plastic bone in the tissue-like liquid that was kept in a plastic bucket [153]. A trapezoidal radiator and truncated ground plane were used to monitor bone fractures in ISM band [154]. The proposed antenna has compact dimensions of $25 mm\times 25 mm\times 0.25 mm$ and uses a Rogers RT/Duroid 5880 substrate. A Vivaldi antenna with standard ($20\times 30\times 1.57$) and miniaturized ($10\times 5\times 1.57$) dimensions was proposed at 1.5 GHz [155] to monitor the bone healing status. Vivaldi type antennas are generally used to provide cross polarization, broadband characteristics, effective radiation patterns, simple feeding methods and ease of fabrication. Table 6 lists some of the antennas that are used in bone implants. Figure 9 shows the implantable antenna used in the literature for bone implant applications.

**Table 6:** Implantable antennas for bone implants

Ref no.	Type of antenna	Dimensions of antenna (mm^3^)	Frequency of operation (MHz)	Bandwidth (MHz)	Gain (dBi)	Substrate used
144	Flexible loop antenna	Cylindrical shape, radius: 5 mm, length: 40 mm	401–406	Not specified	− 27.6	Not specified
145	Monopole antenna	Metal plate: 7 × 2 cm, monopole, radius: 1 mm, length: 3 cm	2000	Not specified	Not specified	Not specified
146	Spiral loop antenna	$2.6 \times 2.6$	2450	20	− 36.2	Rogers RT Duroid 6010), Polydimethylsiloxane
147	UHF antenna	$41 \times 127.81$	860–960	Not specified	1.72	PET
148	Helical antenna	Helical shape, radius: 8.5 mm, length: 30 mm	8000	Not specified	5.91	Not specified
149	Monopole antenna	$30 \times 40$	3000–7000	Not specified	Not specified	Rogers 5880 LZ
150	Spiral split-ring patch antenna	$14 \times 14$	401–406 433–434	68.3	− 36.8 − 35.6	Not specified
151	Planar monopole antenna	$32 \times 30$	2450	Not specified	1.68	FR–4
152	Half-wave dipole antenna	$116 \times 5\times 0.5$	402	Not specified	− 11.29	FR–4
153	Trapezoidal printed	$33.5\times 22\times 1$	2450	Not specified	Not specified	Plastic
154	Trapezoidal	$25\times 25\times 0.25$	4800	1200	3.4	Rogers RT/Duroid 5880
155	Vivaldi	$20\times 30\times 1.57$ $10\times 5\times 1.57$	1500	Not specified	Not specified	FR–4

## Necessity for ongoing research in implantable antennas

The implantable antennas are tiny, resilient communicators embedded inside the human phantom. The following are the essential criteria for unending research on implantable antennas:

- To uncover new materials and coatings that can enhance biocompatibility and minimize the chances of rejection or infection.
- To improve signal strength and dependability within the intricate and variable human body.
- Efficient methods for harvesting energy and wirelessly transmitting it must be developed to ensure the longevity of implantable devices without the need for frequent battery replacements.
- Ensuring the security and privacy of transmitted data is essential as implantable devices become more interconnected to safeguard patient information.
- The integration of implantable antennas with other emerging technologies, such as bioelectronics and nanotechnology, can lead to the development of more sophisticated and versatile medical devices.
- Customizable antennas tailored to individual patient needs and specific medical conditions must be developed to increase the effectiveness of implantable medical devices.

## Conclusion

A review of recent studies has demonstrated that implantable antennas have many possibilities for application across an array of biomedical applications. Real-time data collection and monitoring are made possible by these antennas' ability to provide wireless connections between external monitoring systems and implantable medical devices. Implantable antenna design must consider a number of complications, such as size, efficiency, and biocompatibility. With differing degrees of effectiveness, several antenna types, including patches, helices, meandering line structures, dipoles, and fractal geometries, have been proposed and studied for implantable applications. Implantable antennas have a great impact in medical field, still there are several obstacles to overcome, such as problems with power transfer and frequency distribution. Furthermore, safety issues need to be addressed, especially in light of the possibility of tissue heating and interaction with other electronic equipments. To better understand the potential impact of implantable antennas on human health and to enhance their design and functionality, more research is necessary. In summary, implantable antenna research holds great promise for improving medical monitoring and treatment, making it an intriguing field of study. Although great progress has been made in this field, further study is still needed to solve current issues and ensure both the safety and efficacy of these devices in healthcare environments.

## Data Availability

Not applicable.
